# The associations between students' attitudes toward AI and learning engagement: serial mediating roles of perceived autonomy and learning enjoyment

**DOI:** 10.3389/fpsyg.2025.1681635

**Published:** 2025-12-10

**Authors:** Haiying Liang, Michael J. Reiss

**Affiliations:** 1 School of Foreign Languages, Peking University, Beijing, China; 2 Institute of Education, University College London, London, United Kingdom

**Keywords:** artificial intelligence, learning engagement, perceived autonomy, learning enjoyment, propensity score matching

## Abstract

**Introduction:**

Artificial intelligence (AI) tools are increasingly integrated into higher education to provide real-time feedback and personalized learning support. While previous research has primarily examined the impact of AI attitudes on students' AI usage behavior, less is known about how students' attitudes toward AI influence their psychological experiences and learning behaviors. This study investigates the associations between students' attitudes toward AI and their learning engagement, focusing on the serial mediating roles of perceived autonomy and learning enjoyment.

**Methods:**

A questionnaire was administered to 425 university students. All variables were measured with validated Likert-type scales adapted from established instruments. To reduce common method variance, data were collected at two time points and Harman's single-factor test was performed. Regression analyses, bootstrap mediation testing, and propensity score matching were conducted to examine the proposed serial mediation model and address potential self-selection bias.

**Results:**

A significant positive correlation exists between students' attitudes toward AI and their learning engagement. Perceived autonomy mediates this relationship, with a notable mediation effect of 0.177. Learning enjoyment also plays a mediating role in linking students' attitudes toward AI and their engagement in learning, with a significant and relatively strong mediating effect of 0.115. A serial mediation effect involving both perceived autonomy and learning enjoyment is observed, with a smaller but still significant effect value of 0.021. Furthermore, the use of propensity score matching helps control for self-selection bias, thereby enhancing the robustness of the findings.

**Discussion:**

The findings offer empirical insights into the motivational and emotional mechanisms linking attitudes toward AI and engagement, thereby informing the design of AI-enhanced learning environments to support autonomy, enjoyment, and active participation.

## 1 Introduction

The growing integration of AI tools into educational contexts has sparked increasing interest in how students perceive these technologies and how such perceptions shape their learning experiences. In recent years, AI has become increasingly embedded in academic settings through applications that support writing, summarization, language translation, feedback provision, and content generation (Alqahtani et al., 2023; Zhao, 2024). These technologies are reshaping the way students access information, complete assignments, and interact with learning materials (Chen et al., 2020).

While AI offers clear benefits, it also raises questions about its impact on students' psychological engagement. For instance, excessive reliance on AI may hinder deep processing, reduce effort investment, or create ethical tensions around authorship and academic integrity (Chen et al., 2024; Yusuf et al., 2024). These mixed possibilities make it critical to understand how students feel about AI in learning, and how these attitudes are linked to their motivation, emotions, and learning behaviors.

Prior research has shown that positive attitudes toward AI are closely linked to students' behavioral intentions to adopt these tools (Alzahrani, 2023; Chai et al., 2021; Ma et al., 2024). For example, a recent meta-analysis by Wang and Fan (2025) demonstrated that AI use positively influences students' learning perceptions and satisfaction. However, most existing studies have remained at the level of intention, with relatively few examining whether and how students' attitudes toward AI translate into actual learning engagement—an essential component of academic success.

Learning engagement, a multidimensional construct comprising behavioral, cognitive, and emotional components (Fredricks et al., 2004; Martin, 2007; Pentaraki and Burkholder, 2017), is essential for promoting learning and academic persistence. Although some evidence suggests that AI use may enhance students' learning motivation (Huang et al., 2023; Im et al., 2025; Moybeka et al., 2023), most studies have focused on usage behaviors or self-reported effectiveness, leaving the attitudinal antecedents of learning engagement underexplored. Moreover, Ajlouni et al. (2023) found that students with more positive attitudes toward ChatGPT were more likely to use it in independent learning, suggesting a potential link between students' attitudes toward AI and their learning engagement. Yet, no empirical study to date has systematically tested this direct relationship in a structured model. Therefore, the present study seeks to bridge this gap by examining how attitudes toward AI relate to students' overall learning engagement.

Beyond the direct relationship, understanding how attitudes toward AI translate into learning engagement requires the identification of potential psychological mechanisms. Bognar and Khine (2025) found that students who perceived AI tools as supportive of their learning reported higher levels of academic enthusiasm. Similarly, academic enjoyment has been shown to mediate the effects of motivational and technological variables on engagement (An et al., 2024; Kang and Wu, 2022). Yet, the sequential interplay between autonomy and enjoyment in the context of AI remains untested. According to Self-Determination Theory (Ryan and Deci, 2000), students are more likely to engage in learning when they experience a sense of volition (autonomy) and derive intrinsic satisfaction (enjoyment) from the process. AI tools, when positively perceived, may promote students' sense of autonomy by offering personalized and self-directed learning opportunities (Li et al., 2024; Wu et al., 2024). This in turn may enhance their emotional engagement, as enjoyment is a key affective driver of sustained academic effort (Pekrun and Linnenbrink-Garcia, 2012).

The proposed model suggests that students' positive attitudes toward AI may influence their learning engagement both directly and through two key psychological mechanisms. Favorable attitudes may enhance perceived autonomy by giving students greater control over their learning process, and this heightened autonomy may further promote learning enjoyment. Together, autonomy and enjoyment form a motivational–emotional pathway grounded in Self-Determination Theory, explaining how attitudes toward AI translate into deeper learning engagement.

By modeling autonomy and enjoyment—two key constructs grounded in Self-Determination Theory—as serial mediators, this study aims to illuminate the motivational and emotional processes through which attitudes toward AI affect learning engagement.

This study therefore addresses the following two research questions:

1. To what extent are students' attitudes toward AI associated with their learning engagement?
2. Do perceived autonomy and learning enjoyment sequentially mediate the relationship between students' attitudes toward AI and their learning engagement?

**Figure 1:**
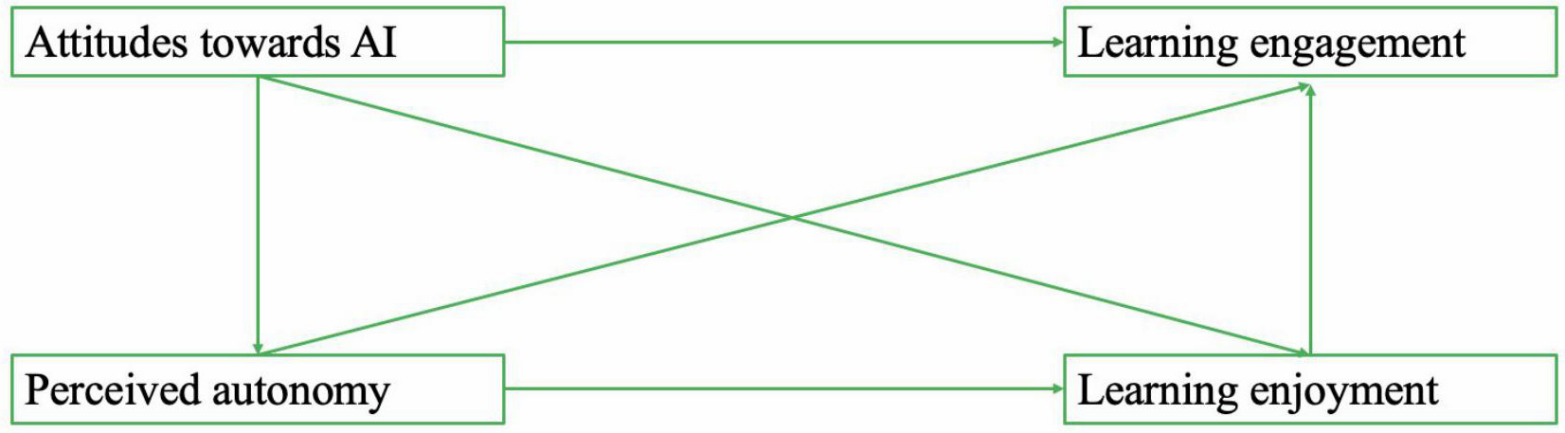
Conceptual model of the proposed mediation pathways.

Figure 1 presents the conceptual framework underlying these proposed relationships.

### 1.1 Research hypotheses

#### 1.1.1 Attitudes toward AI and learning engagement

Learning engagement is a key indicator of students' active involvement in educational activities, encompassing behavioral, emotional, and cognitive dimensions (Skinner et al., 2009). High levels of engagement are often linked to deeper learning, greater academic persistence, and improved academic outcomes. With the increasing integration of AI tools in higher education—such as ChatGPT, Grammarly, and adaptive tutoring systems—students are gaining access to instant feedback, tailored explanations, and a wealth of learning resources (Sajja et al., 2024). These systems can potentially enhance motivation and facilitate more meaningful engagement in academic tasks.

While prior research has demonstrated that AI use is positively associated with learning outcomes (Liang et al., 2023), relatively little is known about the role of students' attitudes toward AI in predicting their level of engagement. Importantly, using AI does not necessarily imply a positive emotional or cognitive orientation toward it; some students may use AI reluctantly or critically, while others embrace it enthusiastically and purposefully. This distinction suggests that students' attitudes—their beliefs, feelings, and behavioral tendencies toward AI—may influence not only whether they use these tools, but also how they engage with learning activities when doing so. Therefore, this research makes the following hypothesis:

*Hypothesis 1:* Students' attitudes toward AI are associated with their learning engagement.

#### 1.1.2 The mediating role of perceived autonomy

Perceived autonomy refers to the extent to which learners feel that their academic behaviors are self-initiated and self-regulated, rather than externally controlled (Deci and Ryan, 1985). Within the framework of Self-Determination Theory (Ryan and Deci, 2000), autonomy is considered a fundamental psychological need that fosters intrinsic motivation, persistence, and meaningful engagement in learning tasks. When students feel autonomous, they are more likely to invest effort voluntarily and experience a greater sense of ownership over their learning process.

In AI-supported learning environments, perceived autonomy may be particularly salient (Niu et al., 2024). Unlike traditional teacher-led instruction, AI tools offer learners greater flexibility in how they access and process information (Chen et al., 2020). For instance, students can decide when to seek assistance, how to engage with instructional content, and to what extent they rely on AI-generated suggestions. This freedom enables them to customize their learning experience, adapt strategies to fit their needs, and progress at a personally appropriate pace. Such individualized control can significantly enhance learners' sense of agency and competence.

Notably, students' attitudes toward AI may play a crucial role in shaping their perceived autonomy. Those who view AI positively are more likely to use it actively and strategically—seeing it as a supportive tool rather than a threat or distraction. As a result, their positive attitudes may increase the extent to which they experience a sense of self-direction and control in the learning process. In turn, this heightened autonomy can promote greater emotional and cognitive engagement. Therefore, this study hypothesizes the following:

*Hypothesis 2:* Perceived autonomy mediates the positive relationship between students' attitudes toward AI and their learning engagement.

#### 1.1.3 The mediating role of learning enjoyment

Learning enjoyment is a positive achievement emotion that arises when learners perceive academic tasks as both valuable and manageable (Pekrun, 2006). As a core component of positive academic affect, enjoyment not only reflects students' emotional engagement but also serves as a powerful driver of motivation, persistence, and deep processing. According to the broaden-and-build theory of positive emotions (Fredrickson, 2001), emotions such as enjoyment can expand students' attention, promote creative thinking, and build long-term learning resources—all of which are closely tied to sustained learning engagement.

AI tools have the potential to enhance learning enjoyment in several ways. First, by providing immediate support and reducing cognitive overload, AI systems can help students feel more competent and less anxious when tackling complex tasks. Second, their interactive and conversational nature—often mimicking peer-like responses—may make learning feel more playful, exploratory, and less intimidating (Almaiah et al., 2022). For example, at least some students may feel more relaxed when posing questions to AI tools like ChatGPT compared to asking instructors, allowing them to experiment, iterate, and learn in a low-stress environment. These emotionally supportive features can create a more enjoyable learning experience, especially for students who struggle with traditional instructional formats.

Crucially, students' attitudes toward AI may influence the extent to which they experience enjoyment. Learners who hold positive views of AI are more likely to perceive its presence as helpful, empowering, and even enjoyable—rather than threatening or impersonal. This positive emotional orientation may lead them to engage more willingly with AI tools, which in turn might be expected to foster a more satisfying and engaging learning experience. Thus, enjoyment may act as a psychological bridge through which favorable AI attitudes enhance student engagement. Accordingly, we propose the following hypothesis:

*Hypothesis 3:* Learning enjoyment mediates the positive relationship between attitudes toward AI and learning engagement.

#### 1.1.4 The serial mediating role of perceived autonomy and learning enjoyment

Perceived autonomy and learning enjoyment are tightly interconnected within motivational and emotion theories. According to Self-Determination Theory (Ryan and Deci, 2000), environments that support learners' sense of autonomy—by offering choice, control, and self-direction—are more likely to elicit intrinsically motivated emotions such as interest, curiosity, and enjoyment. These positive emotions, in turn, have been shown to enhance attention, persistence, and deep-level engagement with academic tasks (Pekrun, 2006).

AI-supported learning environments may naturally facilitate this motivational–emotional sequence. When students hold favorable attitudes toward AI, they are more likely to use it purposefully and flexibly, which enhances their perceived autonomy by allowing them to set their own pace, seek customized feedback, and make independent learning decisions. This increased sense of autonomy can then give rise to greater learning enjoyment, as students feel empowered, less anxious, and more emotionally connected to the task at hand. In this way, AI fosters not just mechanical efficiency, but also a psychologically meaningful learning experience.

This layered process is consistent with prior empirical findings showing that motivational and emotional variables often operate sequentially to explain learning behavior (Linnenbrink and Pintrich, 2003; Pekrun et al., 2011). Specifically, autonomy tends to predict positive emotions, which then contribute to enhanced engagement. Thus, perceived autonomy and learning enjoyment may function as serial mediators linking students' attitudes toward AI with their learning engagement. Accordingly, we propose the following hypothesis:

*Hypothesis 4:* Perceived autonomy and learning enjoyment sequentially mediate the positive relationship between attitudes toward AI and students' learning engagement.

## 2 Methodology

### 2.1 Participants

Data for this study were collected from university students in China using convenience sampling. Questionnaires were administrated via an online platform, Wenjuanxing, which enables nationwide data collection and is commonly employed in behavioral and psychological research. During participant recruitment, we applied screening criteria such as “currently enrolled in undergraduate education” and “aged 18 or above” to ensure alignment with the study's target population. Attention check items (e.g., Please choose the current answer for “which of the following is a fruit”) were embedded to ensure data quality (DeSimone et al., 2015). A total of 473 responses were collected. After removing 31 responses that failed attention checks and an additional 17 responses that deviated by more than three standard deviations (to account for careless or patterned responding), we retained 425 valid responses for analysis.

### 2.2 Attitudes toward AI scale

Students' attitudes toward AI in educational settings were measured using an adapted version of the Attitudes Toward AI Scale developed by Stein et al. (2024). The original scale was validated with strong psychometric properties and was adapted in the present study to fit the learning context. The revised version consists of 12 items covering three dimensions: cognitive, affective, and behavioral. Six items are negatively worded and reverse-scored to reduce acquiescence bias. Responses were recorded on a five-point Likert scale ranging from 1 (strongly disagree) to 5 (strongly agree). The adapted scale demonstrated excellent internal consistency in the current study (Cronbach's α = 0.95).

### 2.3 Learning engagement scale

Learning engagement was assessed using a nine-item version of the scale developed by Reeve and Tseng (2011). Students rated how actively they participated in learning tasks, how absorbed they felt during learning, and how much effort they invested. A sample item is: “I try hard to do well in my learning tasks.” Responses were provided on a five-point Likert scale (1 = strongly disagree, 5 = strongly agree). The scale showed excellent internal consistency (Cronbach's α = 0.96).

### 2.4 Perceived autonomy scale

Perceived autonomy was measured using the autonomy subscale of the Learning Climate Questionnaire (Ryan and Deci, 2000), consisting of six items, adapted to the academic learning context. Participants indicated the extent to which they felt they had control over their learning choices and actions. A sample item is: “I feel free to make my own choices in my learning.” Items were rated on a five-point Likert scale (1 = strongly disagree, 5 = strongly agree). The scale yielded good internal consistency in this study (Cronbach's α = 0.90).

### 2.5 Learning enjoyment scale

Learning enjoyment was assessed using six items adapted from the Academic Emotions Questionnaire (Pekrun et al., 2011), which captures students' positive emotional experiences during academic tasks. Participants rated how much they enjoyed or felt satisfied during their learning activities. A sample item is: “I enjoy the challenge of learning something new.” Responses were provided on a five-point Likert scale (1 = strongly disagree, 5 = strongly agree). The internal consistency of the scale was acceptable (Cronbach's α = 0.85).

### 2.6 Common method variance

To reduce the potential threat of common method variance (CMV), several procedural and statistical remedies were employed in this study, following the guidelines of Podsakoff et al. (2003). First, data were collected across two time points separated by 1 week. In the first wave, participants reported their demographic information and attitudes toward AI. In the second wave, the mediating variables (perceived autonomy and learning enjoyment) and the outcome variable (learning engagement) were measured. This time-lagged design reduced the likelihood of participants forming consistent response patterns based on prior answers.

Second, respondents were assured of the anonymity and confidentiality of their responses and were not informed of the specific research hypotheses. Third, they were clearly instructed that there were no right or wrong answers, and that their participation would bear no academic or personal consequences, encouraging honest and unbiased responses. Fourth, questionnaire items were presented in a randomized order to minimize item-context-induced biases.

Lastly, to examine the potential impact of common method variance (CMV), Harman's single-factor test was performed. The results revealed that the first unrotated factor explained only 19.4% of the total variance in the data. Since this value is well below the commonly accepted threshold of 40%, it indicates that CMV is unlikely to pose a significant threat to the validity of the findings in this study. This suggests that the observed relationships between variables are not unduly influenced by a single underlying factor, thereby reinforcing the robustness of the study's results.

The analytical procedures, including the mediation models and PSM, were cross-checked with an independent statistician to confirm proper implementation and robustness of the findings.

## 3 Results

### 3.1 Descriptive statistics and correlation analysis

The descriptive statistics are presented in Table 1, which outlines the demographic characteristics of the 425 respondents. Among them, 54.6% were male and 45.4% were female. The most common age range for participants was between 22 and 26 years (43.5%). Most respondents were master's students (51.5%), followed by undergraduate (27.8%) and doctoral students (20.7%).

**Table 1:** Demographic information of participants.

**Variable**	**Category**	**Frequency**	**Percentage**
Gender	Male	232	54.6
	Female	193	45.4
Age	18–22	127	29.9
	22–26	185	43.5
	26–30	94	22.1
	Over 30	19	4.5
Education level	Undergraduate	118	27.8
	Masters	219	51.5
	Doctoral	88	20.7
Disciplines	Humanities and social sciences	139	32.7
	Science and engineering	76	17.9
	Business and economics	58	13.7
	Medical and health sciences	106	24.9
	Others	46	10.8

Table 2 presents the descriptive statistics, correlations, and multicollinearity diagnostics for the four key variables: learning engagement, attitudes toward AI, perceived autonomy, and learning enjoyment. As shown in Table 2, all four variables demonstrated moderate to strong positive correlations. Students with more positive attitudes toward AI reported higher levels of perceived autonomy, learning enjoyment, and engagement. Multicollinearity diagnostics indicated no concerns, with all VIF values well below recommended thresholds. These results provide initial support for the expected associations among the study variables.

**Table 2:** Descriptive statistics, correlations and multicollinearity results.

**Variable**	**Mean**	**SD**	**1**	**2**	**3**	**VIF**	**Tolerance**
Learning engagement	2.85	1.15	-			1.478	0.677
Attitudes toward AI	3.09	1.11	0.397	-		1.453	0.688
Perceived autonomy	3.40	1.12	0.557	0.547	-	2.509	0.399
Learning enjoyment	3.55	1.09	0.382	0.338	0.652	1.741	0.574

Learning Engagement has a mean score of 2.85 (SD = 1.15). It shows a significant positive correlation with Attitudes toward AI (*r* = 0.397, *p* < 0.01) and Perceived autonomy (*r* = 0.557, *p* < 0.01). Attitudes toward AI has a mean of 3.09 (SD = 1.11). This variable is moderately correlated with Perceived autonomy (*r* = 0.547, *p* < 0.01) and Learning enjoyment (*r* = 0.382, *p* < 0.01). Perceived autonomy displays a mean score of 3.40 (SD = 1.12) and shows moderate correlations with learning engagement (*r* = 0.557, *p* < 0.01) and Learning enjoyment (*r* = 0.338, *p* < 0.01). Learning enjoyment has a mean of 3.55 (SD = 1.09), and it is moderately correlated with both Attitudes toward AI (*r* = 0.382, *p* < 0.01) and Perceived autonomy (*r* = 0.338, *p* < 0.01).

The multicollinearity diagnostics, including Variance Inflation Factor (VIF) and tolerance, were assessed to ensure the absence of problematic collinearity among the predictors. According to widely accepted guidelines (Hair et al., 2009), VIF values below 5 and tolerance values above 0.2 suggest that multicollinearity is not a concern. As Table 2 shows, all VIF values are below 5, and all tolerance values are greater than 0.2, indicating that multicollinearity does not pose a significant issue in this dataset.

### 3.2 Serial mediating effect

Regression analyses (Table 3) showed that attitudes toward AI significantly predicted learning engagement, and this relationship was reduced but remained significant when the mediators were added, indicating partial mediation. Attitudes toward AI also predicted perceived autonomy and learning enjoyment, and both mediators independently contributed to higher engagement.

**Table 3:** Regression analysis.

**Dependent variable**	**Model 1**	**Model 2**	**Model 3**	**Model 4**
	**Learning engagement**	**Perceived autonomy**	**Learning enjoyment**	**Learning engagement**
Attitudes toward AI	0.315 (*p* < 0.001)	0.144 (*p* < 0.005)	0.240 (*p* < 0.001)	0.141 (*p* < 0.005)
Perceived autonomy			0.199 (*p* < 0.001)	0.233 (*p* < 0.001)
Learning enjoyment				0.145 (*p* < 0.005)
*R* ^2^	0.117	0.180	0.151	0.197
*F*	56.02	46.36	37.51	34.45
*p*	< 0.001	< 0.001	< 0.001	< 0.001

Table 3 summarizes four regression models testing the direct and mediated effects of AI attitudes on engagement.

Hypothesis 1 proposes that students' AI attitudes predict their learning engagement. As shown in Model 1 of Table 3, the coefficient for AI attitudes is significant and positive (β = 0.3146, *p* < 0.001), supporting Hypothesis 1. When the mediating variables are included in Model 4, the coefficient for AI attitudes is reduced (β decreases from 0.3146 to 0.1436, and significance changes from *p* < 0.001 to *p* < 0.005), suggesting that perceived autonomy and learning enjoyment partially mediate this relationship. Regarding perceived autonomy, Model 2 shows that AI attitudes are positively associated with perceived autonomy (β = 0.1436, *p* < 0.005), and Model 3 shows that perceived autonomy significantly predicts learning enjoyment (β = 0.2393, *p* < 0.001), providing support for Hypothesis 2. With respect to learning enjoyment, Model 4 demonstrates that this is a strong positive predictor of learning engagement (β = 0.1445, < 0.005), supporting Hypothesis 3. In addition, Model 3 reveals that AI attitudes also have a significant direct effect on learning enjoyment (β = 0.2396, *p* < 0.001), while Model 4 indicates that perceived autonomy also positively predicts learning engagement (β = 0.2393, *p* < 0.001), which further supports Hypothesis 4 regarding the serial mediation pathway.

To gain a deeper understanding of the magnitude and statistical significance of each mediation pathway, we conducted a more detailed analysis using the Bootstrap method. This approach involved calculating confidence intervals for the indirect effects through repeated resampling, which enabled us to assess the precision and reliability of the estimated mediation effects. By examining whether the confidence intervals for each indirect effect excluded zero, we were able to determine the statistical significance of each mediating pathway. This robust method provides a more accurate estimate of the mediation effects and helps ensure that the findings are not due to random sampling variability. The results of this analysis are presented and summarized in Table 4, offering a clear overview of the significance and strength of each mediation pathway in the study. Bootstrap analyses (Table 4) further confirmed three significant indirect pathways: through perceived autonomy, through learning enjoyment, and through the sequential combination of the two. Together, these findings provide robust support for the proposed parallel and serial mediation model.

**Table 4:** Mediation effect through bootstrap.

**Mediating path**	**Indirect effect**	**Boot standard error**	***P***	**95% CI (lower to upper limit)**	**Relative mediation effect**	**Total mediation effect**
Total effect	0.625	-	0.000	[0.4438, 0.8416]	-	100%
Total indirect effect	0.3125	0.0336	-	[0.2153, 0.4208]	100%	50%
Indirect effect 1	0.1774	0.0246	-	[0.1313, 0.2277]	56.77%	28.39%
Indirect effect 2	0.1146	0.0222	-	[0.0730, 0.1600]	36.67%	18.34%
Indirect effect 3	0.0205	0.0056	-	[0.0110, 0.0331]	6.56%	3.3%

As shown in Table 4, the 95% Bootstrap confidence intervals for all three indirect paths did not include zero, indicating that perceived autonomy and learning enjoyment significantly mediated the relationship between AI attitudes and learning engagement. The total indirect effect was 0.3125, composed of the following three components:

1. Indirect effect 1 (0.1774):
  AI attitudes → perceived autonomy → learning engagement
2. Indirect effect 2 (0.1146):
  AI attitudes → learning enjoyment → learning engagement
3. Indirect effect 3 (0.0205):
  AI attitudes → perceived autonomy → learning enjoyment → learning engagement

**Figure 2:**
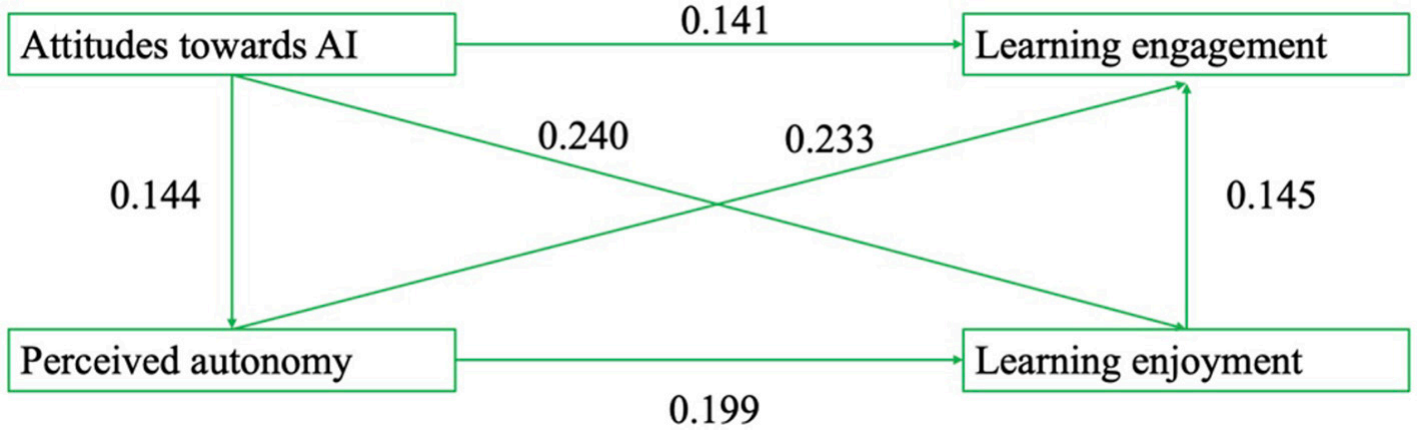
The serial mediation model.

These three effects accounted for 28.4, 18.3, and 3.3% of the total effect, respectively. Together, the results provide robust support for the hypothesized parallel and serial mediation model. Both perceived autonomy and learning enjoyment serve as independent mediators; they also function jointly in a sequential manner, transmitting the effect of AI attitudes on students' learning engagement. A visual summary of these findings is presented in Figure 2.

### 3.3 Potential self-selection bias in mediating models

There may be self-selection bias concerns in the mediating models of this study. For instance, students who hold more positive attitudes toward AI may differ systematically from those with less favorable attitudes in terms of demographic or academic characteristics (such as age, gender, major, etc.) (Rosenbaum and Rubin, 1983). These pre-existing differences could influence their levels of perceived autonomy and learning enjoyment, thus confounding the mediation analysis.

To tackle this potential bias, we implemented the PSM technique, which helps create statistically comparable treatment and control groups. The fundamental principle of PSM, as outlined by Rosenbaum and Rubin (1983), involves matching each participant in the treatment group with one or more individuals in the control group who share similar observed characteristics, such as age, gender, academic discipline, and other relevant demographic factors. In this research, students with more positive attitudes toward AI may systematically differ from those with less positive attitudes in terms of demographic characteristics or psychological attributes. Such pre-existing differences can introduce self-selection bias, meaning that simple regression analyses may not fully isolate the effect of AI attitudes from confounding background factors. PSM helps address this issue by creating statistically comparable groups of students with high and low AI attitudes based on observed covariates. This procedure reduces imbalance between groups and provides a more robust estimate of the associations between AI attitudes, perceived autonomy, and learning enjoyment than regression alone. By pairing students with similar propensities, PSM reduces the impact of confounding variables that could distort the treatment effects. This process enhances the validity of our findings by ensuring that any differences between the groups can be attributed more confidently to the treatment itself, rather than to pre-existing differences between participants.

In this study, participants were assigned to either a treatment or control group based on their scores on the Attitudes Toward Generative AI Scale (Q6–Q17). Specifically, those with scores above the sample median were placed in the treatment group (*G*_*i*_ = 1), while those with scores below the median were placed in the control group (*G*_*i*_ = 0). To ensure a clear conceptual distinction between groups, participants whose scores were exactly equal to the median (*n* = 27) were excluded from the analysis. This resulted in two groups of equal size, with 199 participants in each group.

To estimate the average treatment effect on the treated (ATT**)**, we compared the outcomes—perceived autonomy and learning enjoyment—between the two matched groups. The outcome variables are denoted as Output_i1_ for students in the treatment group and Output_i0_ for those in the control group. The ATT is defined as:

*t*_*ATT*_ = *E* (Output_i1_ | G_*i*_ = 1) – *E* (Output_i0_ | *G*_*i*_ = 1)

Because the counterfactual *E* (Output_i0_ | *G*_*i*_ = 1) is unobservable, we approximate it using the average values from matched students in the control group who share similar covariates. The output variables are denoted as Output_i1_ for the treatment group and Output_i0_ for the control group. Let Output_i1_ denote the observed outcome variable (e.g., perceived autonomy or learning enjoyment) for participants in the treatment group, and Output_i0_ denote the counterfactual outcome had those same individuals not had high AI attitudes. Since *E* (Output_i1_ | G_*i*_ = 1) is unobservable, we approximate it using the average outcome of students in the control group who share similar observed characteristics. Thus, the ATT can be estimated as:

*t*_*ATT*_ = *E* (Output_i1_ | G_*i*_ = 1) – *E* (Output_i0_ | *G*_*i*_ = 0)

This estimation assumes that the matched students in both groups are similar in terms of key covariates. Following Villalonga (2004), we estimated propensity scores as the probability of a student being in the treatment group conditional on a set of observed covariates X_*i*_:

*p*(X_*i*_) = Pr (G_*i*_ = 1 | X_*i*_) = E (G_*i*_ | X_*i*_)

**Figure 3:**
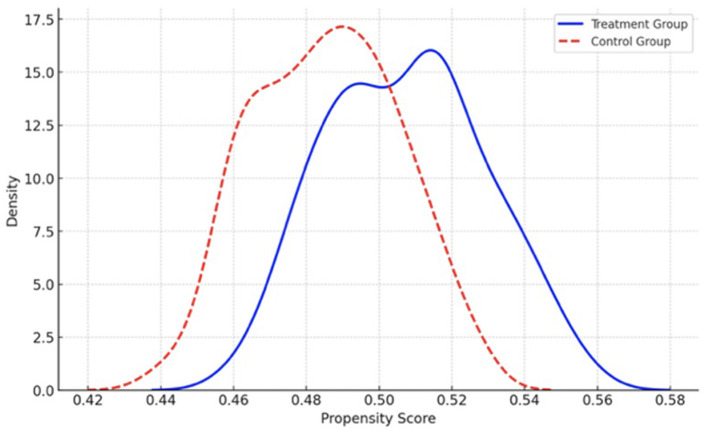
The graph of kernel density functions of treatment and control groups before matching.

**Figure 4:**
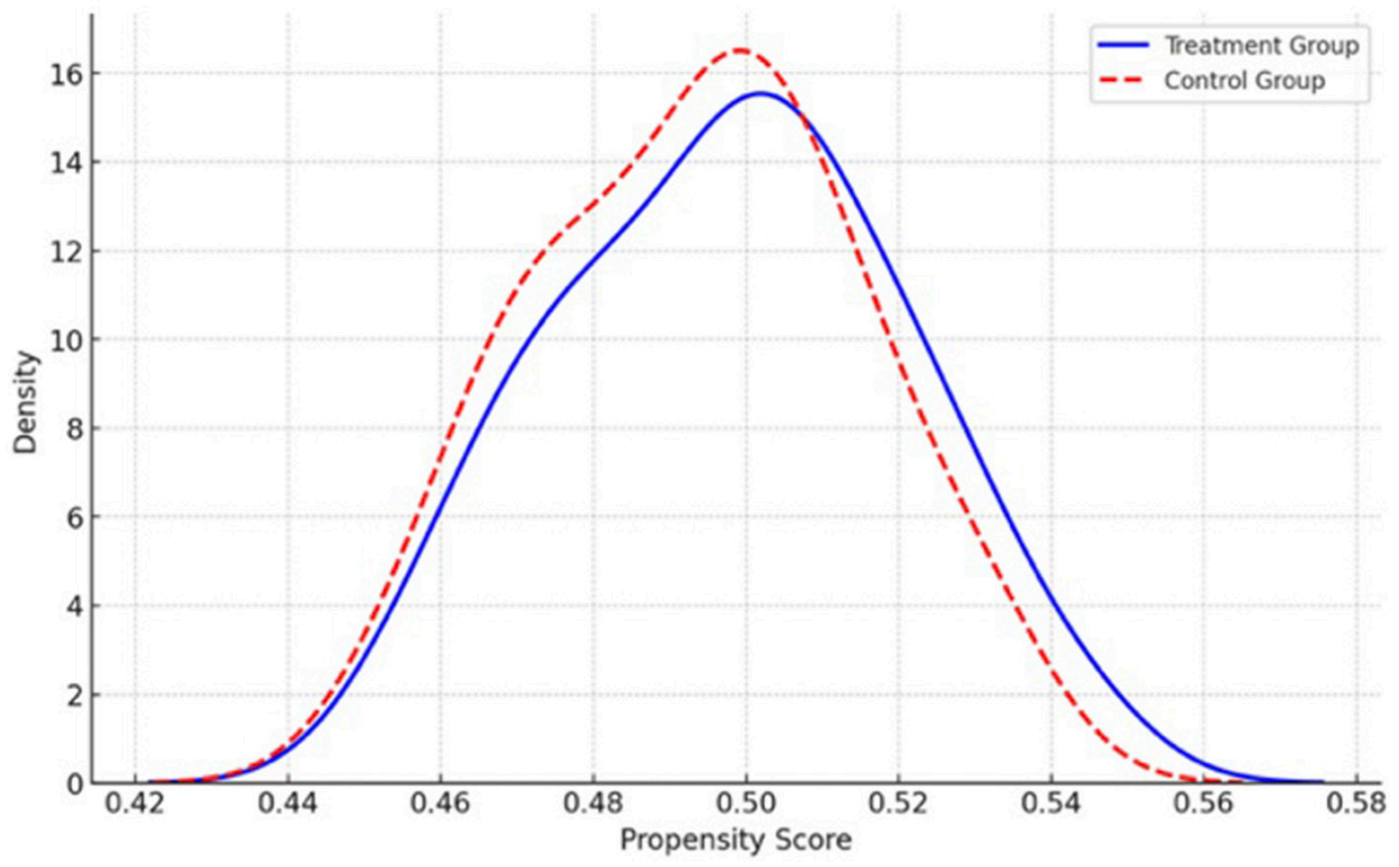
The graph of kernel density functions of treatment and control groups after matching.

In this study, vector X_*i*_ represents the characteristics that may influence the outcome variables, such as the participant's age and gender. To control for potential confounding variables, we applied the nearest neighbor matching method to align the treatment and control groups based on their estimated propensity scores. These scores were derived using a probit regression model, which allowed us to match participants in the two groups with similar covariates, thereby reducing selection bias and ensuring a more accurate comparison of the treatment effects. The kernel density distributions of propensity scores for both groups are shown in Figures 3, 4.

As shown in Figure 3, the kernel density functions for the treatment group (students with high AI attitudes) and the control group (students with low AI attitudes) exhibit a noticeable discrepancy before matching, highlighting an imbalance in the observed covariates. However, after the matching process (Figure 4), the distributions of the two groups converge, demonstrating that the matching procedure has successfully aligned the treatment and control groups. This improvement in the distribution suggests that, after matching, the groups are more comparable with respect to their individual characteristics, reducing the potential for confounding effects and ensuring a more valid comparison between the groups.

Based on the matched sample, the treatment effect estimates for the outcome variables—perceived autonomy and learning enjoyment—are presented in Table 5. Results from Table 5 show that the average levels of both perceived autonomy and learning enjoyment are significantly higher among students in the treatment group compared to those in the control group. The estimated ATT values are positive and statistically significant. These findings suggest that students with more positive attitudes toward AI tend to experience higher levels of perceived autonomy and learning enjoyment, even after controlling for self-selection bias.

**Table 5:** PSM results.

**Treatment variable**	**Output variable**	**Sample**	**Average value of output variable for the treatment group**	**Average value of output variable for the control group**	**ATT (average treatment effect on the treated)**	***t*-stat**
Attitudes toward AI	Perceived autonomy	Unmatched	3.72	3.14	0.158	3.71
		Matched	3.71	3.24	0.147	2.34
Attitudes toward AI	Learning enjoyment	Unmatched	3.78	3.32	0.145	3.67
		Matched	3.77	3.35	0.142	2.14

## 4 Discussion

### 4.1 Theoretical implications

This study provides new insights into the relationship between students' attitudes toward AI and their learning engagement, demonstrating a significant positive correlation between the two. Previous research has predominantly focused on the direct impact of AI tools on academic performance, often examining how AI affects learning outcomes in terms of grades or achievement (Eltahir and Babiker, 2024; Liang et al., 2023). However, the psychological factors driving these outcomes—such as students' attitudes toward AI—have been largely underexplored. By examining the role of students' emotional and cognitive reactions to AI, this study extends the existing body of literature, highlighting that positive AI attitudes are not only linked to the adoption of AI tools but also to deeper engagement with learning activities. This finding supports the notion that students' psychological alignment with AI technologies plays a crucial role in fostering sustained academic effort, an idea that aligns with earlier studies on technology acceptance in educational settings (Altememy et al., 2023; Lin and Chen, 2024).

In addition, by demonstrating the serial mediating roles of perceived autonomy and learning enjoyment, this study introduces a new mechanism through which AI attitudes influence learning engagement. Previous studies have shown that learning enjoyment may enhance learning engagement (Liu, 2022; Wang, 2022; Zhao and Yang, 2022), while other studies have found that students' perceptions of learning autonomy increase learning enjoyment (Hagensauer and Hascher, 2010; Hinnersmann et al., 2020; Zhang et al., 2022). This research supports the serial mediation pathway, suggesting that when AI tools are perceived positively, they can sequentially enhance students' sense of autonomy and enjoyment, which in turn boosts their engagement with learning. By positioning perceived autonomy and learning enjoyment as sequential mediators, this study expands on existing research into the emotional and motivational processes underlying students' academic engagement.

Beyond aligning with established principles of Self-Determination Theory, the present findings contribute new theoretical insights by demonstrating that autonomy and enjoyment operate in a sequential manner in AI-enhanced learning environments. While prior Self-Determination Theory research typically examines motivational and emotional constructs in parallel, our results suggest that AI tools may trigger a dynamic process in which perceived autonomy first enables learners to feel more in control of their learning decisions, which subsequently enhances their enjoyment. This sequential pathway sheds light on how AI functions not only as a cognitive aid but also as a catalyst for internal motivational transformation. By identifying this autonomy-to-enjoyment mechanism, the study extends Self-Determination Theory into the context of generative AI and illustrates how psychological needs can be fulfilled in technologically mediated learning environments.

### 4.2 Practical implications

#### 4.2.1 The impact of students' attitudes toward AI on learning engagement

This study underscores the importance of students' attitudes toward AI in shaping their learning engagement. The results show that students with positive attitudes toward AI are more likely to engage with their learning. This finding contributes to a growing body of research suggesting that technology's impact on education goes beyond mere usage and is also influenced by how students perceive and relate to it (Chan and Hu, 2023; Strain-Moritz, 2016).

When students feel free to make choices and find the learning process enjoyable, they are more likely to stay engaged. Designing flexible and enjoyable AI-based tasks can therefore strengthen students' motivation and participation, as suggested by Sajja et al. (2024).

#### 4.2.2 The mediating roles of perceived autonomy and learning enjoyment

The findings from this study also highlight the critical role that perceived autonomy and learning enjoyment play in mediating the relationship between students' attitudes toward AI and their learning engagement. Specifically, we found that students who feel more autonomous in their use of AI tools—and who derive enjoyment from interacting with these tools—are more likely to be engaged in their learning. This underscores the importance of psychological and emotional factors in fostering learning engagement.

Perceived autonomy, grounded in Self-Determination Theory (Ryan and Deci, 2000), emerges as a central mediator. When students feel that they have control over how they use AI—whether it's choosing which AI tools to engage with or determining the pace of their learning—they are more likely to invest effort in the learning process. The freedom to make learning decisions enhances their sense of ownership and motivation, which leads to higher engagement levels. Educators can capitalize on this by designing learning environments where students can personalize their use of AI, giving them autonomy to explore different tools and learning pathways.

In parallel, learning enjoyment emerges as another key mediator in this process. As Pekrun (2006) suggests, when students enjoy their learning experience, they are more likely to maintain interest and invest effort in academic tasks. Positive attitudes toward AI can enhance this emotional experience, as AI tools that offer interactive, feedback-driven, and personalized learning experiences are more likely to foster enjoyment. This emotional connection to the learning process, when paired with a sense of autonomy, creates a powerful motivational force that drives students to engage more deeply with the material. By designing learning tasks that are not only intellectually stimulating but also enjoyable, educators can further amplify both cognitive and emotional engagement.

The serial mediation model, where perceived autonomy enhances learning enjoyment, which in turn increases engagement, provides a nuanced understanding of how AI tools can foster meaningful learning experiences. This model highlights the interconnectedness of motivation and emotions in the learning process, as proposed by Pekrun et al. (2011). When students feel empowered by AI to make decisions about their learning and enjoy the process, they are more likely to engage deeply with the tasks at hand.

### 4.3 Limitations

While this study provides valuable insights into the relationship between students' attitudes toward AI and their learning engagement, several limitations must be acknowledged. First, the study employed a cross-sectional design, which limits the ability to draw causal inferences about the relationships between the key variables. Although the findings suggest significant associations, future research with a longitudinal or experimental design could provide a clearer understanding of the causal directionality between AI attitudes, perceived autonomy, learning enjoyment, and learning engagement.

Second, it is important to note that the findings of this study are situated within the cultural and educational context of China, where AI technologies have been rapidly promoted in higher education and widely integrated into teaching and learning practices. Chinese university students are generally accustomed to technologically mediated learning environments, and national educational policies have strongly encouraged the adoption of AI to support personalized learning and improve academic efficiency. These factors may contribute to more positive attitudes toward AI and a stronger perception of AI as a useful learning companion. At the same time, the exam-oriented nature of Chinese education may influence the ways students use AI tools, particularly in relation to autonomy and enjoyment.

While the underlying psychological mechanisms proposed in this study—autonomy, enjoyment, and engagement—are grounded in universal principles of Self-Determination Theory, the strength of the relationships may vary across cultural contexts. For example, students in educational systems with different levels of technological adoption, instructional autonomy, or attitudes toward academic integrity may respond differently to AI tools. Future cross-cultural studies are therefore needed to examine whether similar patterns hold in different cultural and institutional environments.

Third, while this study focused on attitudes toward AI, it did not account for individual differences in technology literacy or prior experience with AI that may influence students' engagement with AI tools. These factors could potentially moderate the relationships observed in this study. Future studies could explore how such individual differences impact learning engagement and how AI tools can be tailored to meet diverse student needs.

Finally, the study relied on self-reported data, which may be subject to biases such as social desirability or recall bias. Although efforts were made to minimize these biases through careful questionnaire design, the use of objective measures, such as behavioral data or instructor assessments, in future studies could provide a more comprehensive understanding of how AI attitudes influence learning engagement.

## 5 Conclusion

This study explores the impact of students' attitudes toward AI on their learning engagement, emphasizing the mediating roles of perceived autonomy and learning enjoyment. The findings indicate that positive attitudes toward AI are significantly associated with higher levels of learning engagement, with perceived autonomy and learning enjoyment acting as key mediators in this relationship. These results align with Self-Determination Theory, suggesting that when students perceive AI as a tool that enhances their autonomy and provides an enjoyable learning experience, they are more likely to engage deeply with their learning tasks.

The study contributes to the growing body of literature on AI in education by providing empirical evidence of the psychological mechanisms through which students' attitudes toward AI influence their learning behaviors. The serial mediation model, where perceived autonomy enhances learning enjoyment, which in turn promotes engagement, offers a nuanced understanding of the motivational and emotional processes underlying AI use in academic settings.

Practically, the findings underscore the importance of fostering positive attitudes toward AI among students and designing learning environments that promote both autonomy and enjoyment. Educators and instructional designers should focus on creating tasks that allow for personalized learning experiences, encourage creative exploration, and enhance students' emotional engagement with the learning process.

While this study provides valuable insights, future research is needed to further explore the causal relationships between AI attitudes and learning engagement, as well as to examine the influence of individual differences and cultural contexts. Overall, this study lays the groundwork for future investigations into the role of AI in education, offering practical guidance for integrating AI tools into teaching and learning strategies to enhance student engagement and motivation.

## Data Availability

The raw data supporting the conclusions of this article will be made available by the authors, without undue reservation.
